# The Groot Effect: Plant facilitation and desert shrub regrowth following extensive damage

**DOI:** 10.1002/ece3.3671

**Published:** 2017-12-05

**Authors:** Christopher J. Lortie, Eva Gruber, Alex Filazzola, Taylor Noble, Michael Westphal

**Affiliations:** ^1^ Department of Biology York University Toronto ON Canada; ^2^ Department of Wildlife Humboldt State University Arcata CA USA; ^3^ Bureau of Land Management Central Coast Field Office Marina CA USA

**Keywords:** clipping, damage, deserts, facilitation, positive interactions, shrubs

## Abstract

Deserts are increasing in extent globally, but existing deserts are decreasing in health. The basic biology and ecology of foundation plant species in deserts are limited. This is a direct study that provides an estimate of the capacity for a locally dominant foundation shrub species in California to recover from damage. Desert shrubs are cleared and damaged by humans for many purposes including agriculture, oil and gas production, and sustainable energy developments; we need to know whether foundation species consistently facilitate the abundance and diversity of other plants in high‐stress ecosystems and whether they can recover. A total of 20 *Ephedra californica* shrubs were clipped to the ground at a single site and systematically resampled for regrowth 2 years later. These shrubs were damaged once and regrew rapidly, and relatively, larger shrubs were not more resilient. This study provides evidence for what we termed the “Groot Effect” because smaller individuals of this shrub species can recover from significant aboveground damage and continue to have positive effects on other plant species (similar to the popular culture reference to a benefactor tree species). The density of other plant species was consistently facilitated while effects on diversity varied with season. These findings confirm that *E. californica* is a foundation species that can be an important restoration tool within the deserts of California in spite of extreme cycles of drought and physical damage to its canopy.

## 1 INTRODUCTION

Shrubs frequently function as foundation species within desert ecosystems. A foundation species in ecology is defined as one with significant and often singular impacts on the structure and sometimes functioning, of an ecosystem (Angelini, Altieri, Silliman, & Bertness, 2011). The effects of a foundation species in any given system are varied (Ellison et al., 2005), change with context (Angelini et al., 2011; Malatesta, Tardella, Piermarteri, & Catorci, 2016; Sthultz, Gehring, & Whitham, 2007), and can influence the range and niche for other species through positive interactions or facilitation (Bruno, Stachowicz, & Bertness, 2003; Bulleri, Bruno, Silliman, & Stachowicz, 2016). In deserts, shrubs can influence other plant species through numerous mechanistic pathways including seed trapping, shelter, refuges from predation, and environmental amelioration (Filazzola & Lortie, 2014). The canopy of shrubs is often assumed to be the primary structural agent of facilitation for plants and animals through direct and indirect shelter and refuge effects (Badano et al., 2016; Brathen & Lortie, 2016). Understanding the functional ecology of foundation plant species through traits such as the canopy can improve our capacity to estimate sensitivity of positive interactions to global changes including disturbance, fire, physical damage, and drought.

Deserts expand, and their health declines. Global desertification is a pressing issue because of arid expansion (Asner & Heidebrecht, 2005; Kefi et al., 2007). Global dryland science typically focuses on loss of semiarid grasslands to shrubs (Reynolds et al., 2007; Torres, Abraham, Rubio, Barbero‐Sierra, & Ruiz‐Pérez, 2015) with less attention on mechanisms associated with healthy desert functioning from the shrubs. Prolonged drought and increasing variability in precipitation and temperature (Kogan & Guo, 2015; MacDonald, 2007) likely impact shrub function as foundation species in these ecosystems (Madrigal‐González, Kelt, Meserve, Squeo, & Gutiérrez, 2016; Reed & Loik, 2016). There is research on the regrowth patterns of shrubs following fire within arid and semiarid ecosystems (Clarke & Knox, 2002) and limited research on the effects of drought on woody regeneration (Martínez‐Vilalta & Lloret, 2016). Importantly, desert shrub regrowth following aboveground physical damage, from browsing for instance, can take a number of years and be incomplete (da Silveira Pontes, Magda, Gleizes, & Agreil, 2016). Studies of canopy regrowth patterns following mechanical damage are thus a viable research bridge between form and (ecological) function and provide evidence for a novel research gap in desert foundation species biology.

The purpose of this specific study was to directly examine whether a common desert shrub species within California, *Ephedra californica*, can recover from mechanical damage introduced during a period of extended drought. Consequently, the general hypothesis tested was that even with substantial mechanical damage via branch removal in a drought year and through persistent interannual drought, a common shrub species can recover via resprouting and that size confers resilience. The following predictions were tested (1) *E. californica* is a foundation plant species in one region of California because it facilitates other plant species, (2) a desert shrub species can significantly recover from a major physical damage event, and (3) larger shrubs (estimated by canopy volume) significantly recover more than relatively smaller individuals. Damaging desert shrubs is not ideal, and we do not advocate shrub destruction as a routine research action. Nonetheless, it is important to explore the extent that a canopy recovers following controlled damage to understand the capacity for recovery and ecological function.

## 2 METHODS

### 2.1 Study species

The focal shrub study species was *E. californica* known as Mormon Tea (Hickman, 1996). It is in the Gnetales division reaching heights of approximately 1 m or more (Cutlar, 1939), and it is native to California and Baja Mexico and widely distributed (Hollander, Wall, & Baguley, 2010; Villanueva‐Almanza & Fonseca, 2011). It is a profusely branching shrub species with unique characteristics of both angiosperms and gymnosperms possessing numerous twigs and tiny leaf‐life needles at its axial nodes (Alfieri & Mottola, 1983). This makes it an ideal species, morphologically, to study canopy regrowth and examine size‐specific sensitivities. This shrub species is common at the study site described below (Filazzola et al., 2017; Hawbecker, 1951).

### 2.2 Study site

The study was performed at the Panoche‐Coalinga Area of Environmental Concern administered by the US Bureau of Land Management (36°41.776′N, 120°47.886′W at 650 m. a.s.l.). This site is within the San Joaquin Desert and is best described as a large, distributed and highly impacted/fragmented desert ecosystem (Germano et al., 2011). Research was performed on a relatively flat plateau situated above the main floor of the San Joaquin Valley. The climate is arid to semiarid with rainfall in winter months. Temperatures between 10°C and 30°C during the plant‐growing season January to July, and the 20‐year long‐term mean annual precipitation for region is 153 mm (Panoche Weather Station data at 37°03.30′N, 120°51.00′W from U.S. Climate Data 2017, Appendix S1). This region and specific site have experienced both a severe and extended “mega‐drought” from 2006 to 2015 (Kogan & Guo, 2015), and experimental surveys and manipulations began within this period of high stress (Appendix S1) and into the 2016–2017 growing season.

### 2.3 Experimental design

To examine foundation effects of *E. californica* on other plant species, a total of 225 shrubs were surveyed annually at the end of the growing seasons from 2013 to 2016 inclusively. The longest diameter of the shrub canopy, a second measure of diameter perpendicular to the maximum, and height were recorded annually for every shrub. The volume of each shrub was estimated using the formula for a semisphere. In each instance, a 0.5‐m quadrat was placed under the canopy within the drip line, and total annual abundance and plant species richness were recorded. Using the quadrat, an estimate of annual vegetation within the “open” was similarly recorded, paired with and within 1 m of every shrub. Open was defined as annual vegetation only, not within the drip line of any shrub, and also at least 0.5 m from another shrub. These adjacent open pairs were systematically selected in each instance to meet these criteria. Shrubs were selected at random within the region to ensure a representative sample of shrub sizes and microtopographic variation.

In 2014 at the end of the winter growing season, a total of 20 individual *E. californica* shrubs were clipped to the ground and all aboveground biomass including litter was removed. Similar to the regional survey of shrubs, the diameter in longest and perpendicular axes was measured in addition to height prior to clipping. In December of 2016 after first rains, these individual shrubs were censused for regrowth. The volumetric size dimensions were recorded in the field, and the total number of basal shoots was also counted and classified as resprouted shoots (Cornelissen et al., 2003). The shrubs were then clipped anew, to the ground. All aboveground biomass was retained in this instance and weighed wet. The shrub *E. californica* expresses significant axillary (i.e., secondary) branching (Appendix S2 for photograph).

### 2.4 Statistical analyses

All analyses were performed using R version 3.3.2, and full data and statistical workflows are available here https://cjlortie.github.io/ephedra.recovery/. The prediction that *E. californica* functions as a foundation species for other plants was estimated using the relative interaction intensity effect size measure (Rii) (Armas, Ordiales, & Pugnaire, 2004). Linear‐mixed models with a Satterthwaite approximation for degrees of freedom were used to test for global effect of shrubs on association patterns of annuals relative to the open using the package “lmerTest” (Brockhoff, 2016). The function “lmer” was used with year as a random effect and shrub volume modeled as covariate. The *p*‐value for the random effect year was estimated using the “rand” function from this package and model “summary” function. Post hoc, one‐sample *t* tests were used to test whether the mean Rii effect size estimate was significantly different from μ* *= 0 in each year. An ancillary simple test of net effects was also carried out because effect size measures are simplified and derived data; these were one‐sample *t* tests if the *E. californica* grand mean values were different from no effect pooled across the entire duration of the experiment. Richness is the total number of unique annual plant species within plots, and abundance is the total number of individual annual plants. Linear‐mixed models with a Satterthwaite approximation for degrees of freedom were similarly used to test for differences in shrub volumes and height with year as a random effect with appropriate post hoc tests on the effect size measures. Probability density functions for regrowth patterns after two years in 2016 were plotted and estimated using the “geom_density” and “ggplot_build” functions from the “ggplot2” package using Gaussian kernels (Wickham, 2016). A density plot is a smoothed histogram using the conditional probabilities for the density intervals or binwidths (Hall, Racine, & Li, 2004). Post hoc regression analyses were used to test for shrub size‐dependent effects on recovery following damage. Linear versus curvilinear fits were contrasted using AIC scores (Aerts, Claeskens, & Hart, 2000; Hurvich & Tsai, 1989). Residuals were inspected using Normal Q‐Q and fitted versus predicted plots using the “stats” package (R‐Core‐Team, 2016), and outliers were examined using chi‐squared tests from the “outliers” package in R (Komsta, 2015). Quantile regressions were used as a secondary test for size relationships when explained variation in linear regressions was relatively low (i.e., *r* ^2 ^< 20%) and when unequal variation patterns were likely due to complex interactions associated with different sized individual shrubs response variables (Cade & Noon, 2003). These regressions were carried out using the “quantreg” package with a Hall–Sheather bandwidth rule (Koenker, 2017).

## 3 RESULTS

**Figure 1:**
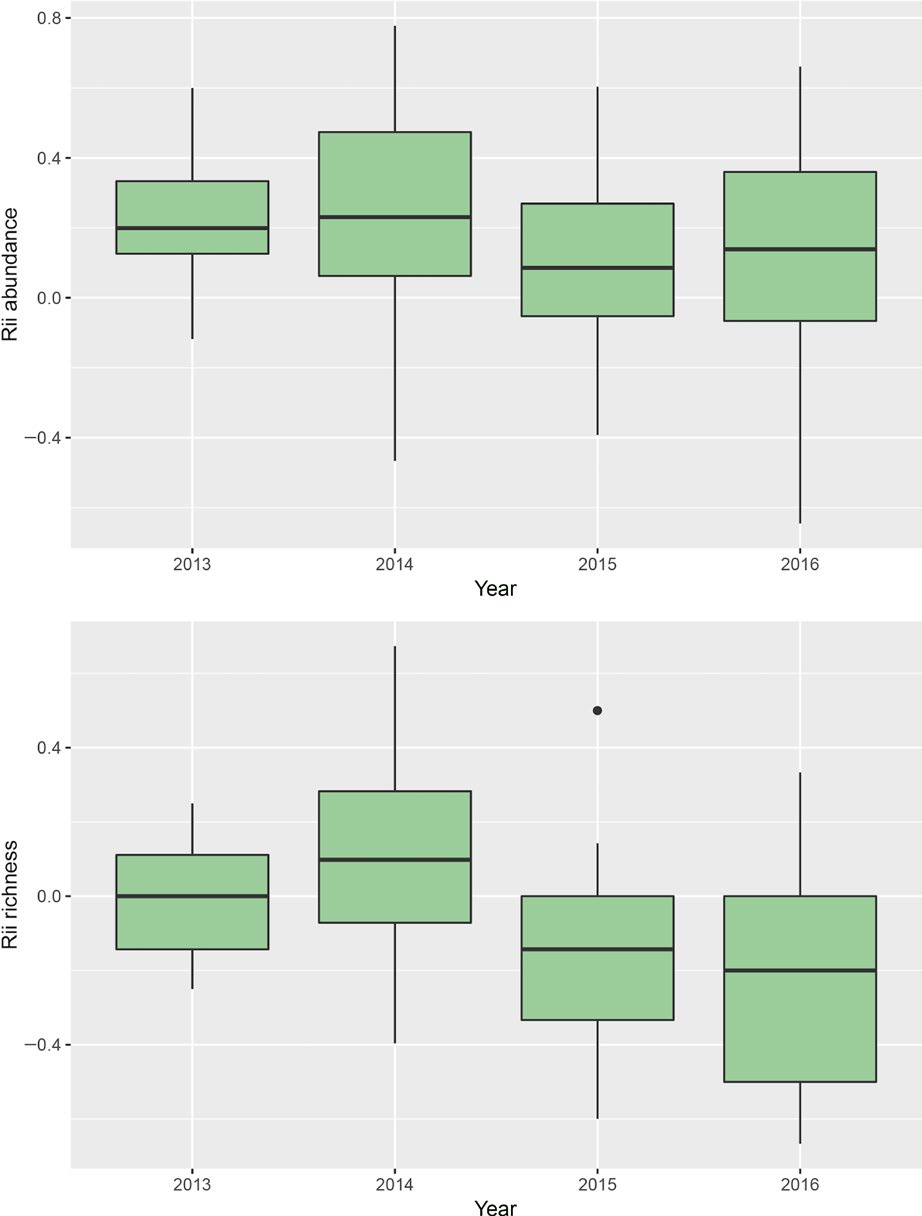
The relative association patterns of annual plants within a desert ecosystem, Panoche Hills Ecological Reserve, between paired shrub and open vegetation surveys. The shrub species tested was *Ephedra californica*, and plots were 0.5 m in size and square and placed under shrubs and at paired, adjacent open sites within a canopy at distances of up to 1.5 m from a shrub. The relative interaction intensity effect size measure (Rii) is reported with positive values denoting positive associations or facilitation and negative values denoting negative associations (bounded from −1 to +1). The solid middle line in boxplots shows the median of data, whiskers show 1.5 standard deviations, and dots show outliers >1.5 quantile. Abundance is total number of individual annual plants within plots, and richness is the cumulative mean total of unique plant species

The relative abundance and richness of the plants within this region significantly varied between *E. californica* and paired open sites (Figure 1, Table 1) and were also significantly different from no net effect of the shrubs (one‐sample *t* tests on grand means, abundance, *t* = 8.6, *df* = 222, *p* = .0001, richness, *t* = −6.2, *df* = 222, *p* = .0001). The abundances of annual plants were consistently and positively associated with this shrub species in all years (Figure 1 with one‐sample *t* test post hoc contrasts), while the richness of the annual plant community varied significantly from neutral (i.e., not significantly different from 0 or no net effect in one‐sample *t* test post hoc contrasts) to positive or negative depending on the growing season (Figure 1). Relatively larger shrubs did not have significantly greater (or consistent) effects on the net outcome of interactions with the annual plant community (Appendix S3).

**Table 1:** Global linear‐mixed models to test for effects of *Ephedra californica* shrubs on the relative abundance of species richness of annual plants within this system

Measure	Effect	Estimate	*df*	*p*‐value
Rii (abundance)	Intercept	0.2	4	**.006**
	Volume	0.29	220	.05
	Year	3.58	1	.06
Rii (richness)	Intercept	−0.1	3	.26
	Volume	0.0008	0.002	.09
	Year	54.5	1	**.0001**

Linear‐mixed models with a Satterthwaite approximation for degrees of freedom were used with year as nested, random effect and volume as a covariate. The global models were used to identify large net differences in the Rii effect size measure (see text for description). Bold values highlight alpha at *p* < 0.05.

**Figure 2:**
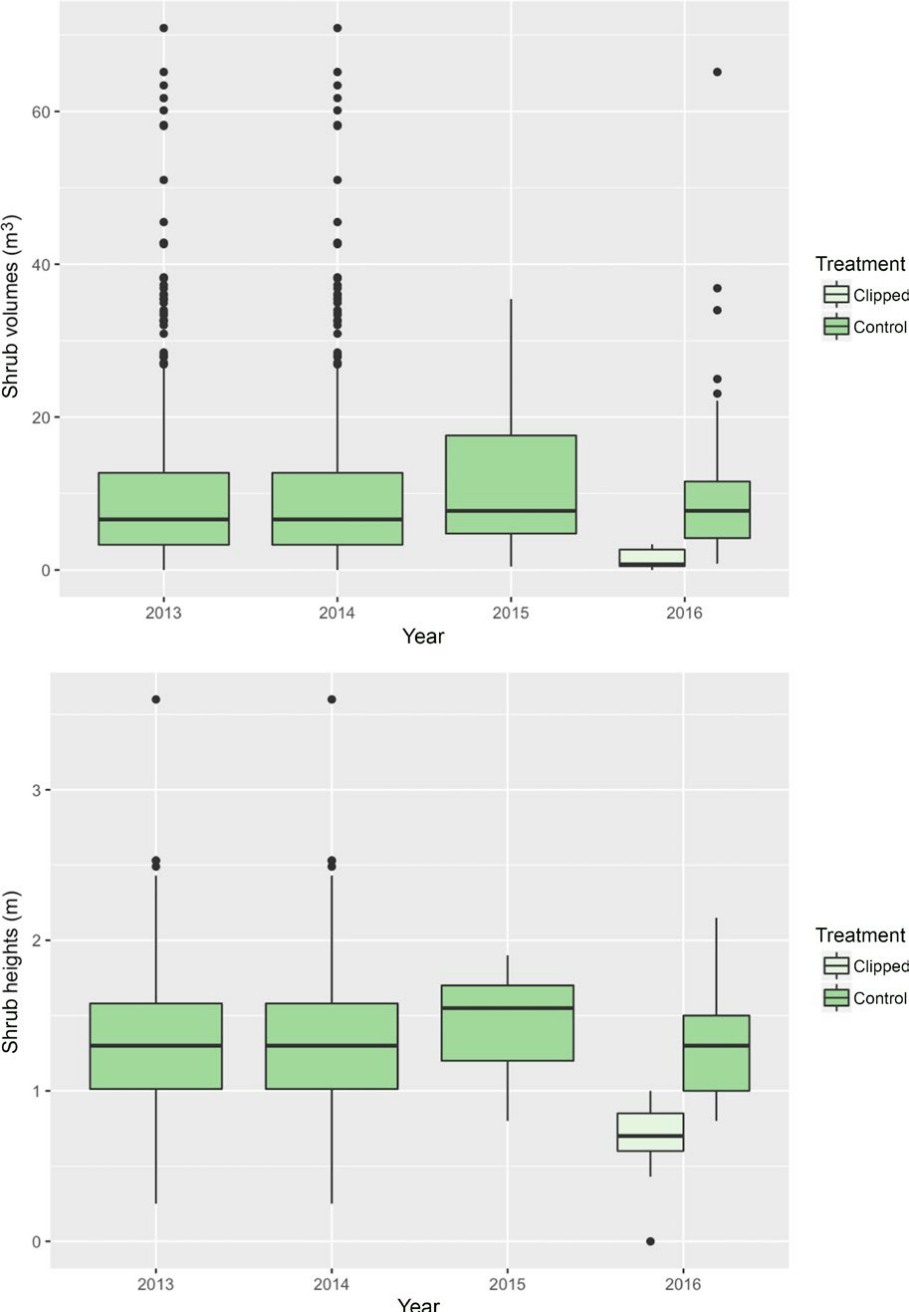
The interannual effect of aboveground clipping and removal of biomass on the canopy and height of the desert shrub *Ephedra californica*. The shrubs were surveyed in 2013 during the growing season and clipped the following growing season. The first census of regrowth was in 2014. See Methods for calculation of shrub volumes. The solid middle line in boxplots shows the median of data, whiskers show 1.5 standard deviations, and dots show outliers >1.5 quantile

**Figure 3:**
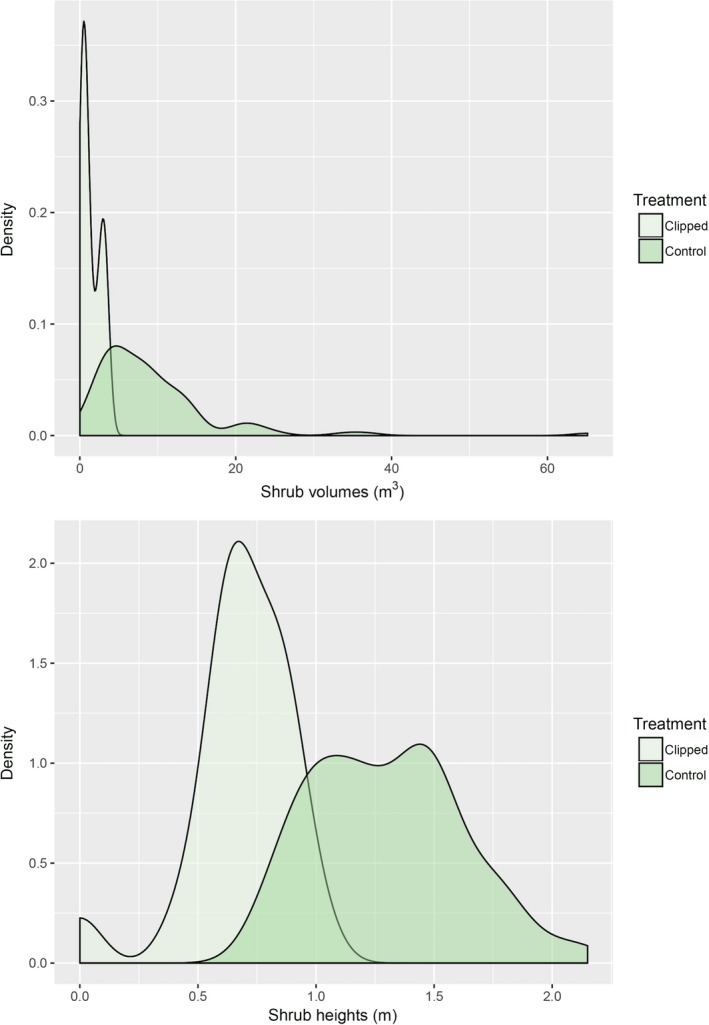
Smoothed density estimate plots for clipped and control *Ephedra californica* shrubs in 2016 after 2 years of regrowth. Volume refers to canopy volume estimated using height and width measures (see text for calculations), and height is to the highest vertical living stem of a given shrub. The density estimates show the probability of those measures over the specific intervals listed on the *x*‐axis fit to a Gaussian kernel distribution (see statistical methods for detailed description in text)

Clipping the shoots of shrubs and removing all biomass significantly reduced the canopy shrub volumes and heights (Figure 2, Table 2). There was no difference between the treatment and control shrub volumes or heights prior to clipping at the onset of experiment but significant differences in each of the years subsequent to the treatment (Figure 2 with Tukey's post hoc contrasts). While the clipped and control shrubs significantly differed in volume and heights at the end of the 2016 growing season (Figure 2), there was nonetheless significant regrowth because the upper and lower quantiles of the estimated probability distributions for volume and heights overlapped (Figure 3, smoothed density probability estimates using a Gaussian kernel distribution).

**Table 2:** Linear‐mixed models to test effects of aboveground clipping of *Ephedra californica* shrubs on canopy regrowth

Measure	Effect	Estimate	*df*	*p*‐value
Shrub volumes	Treatment	44.4	1	.0001
	Year	2.2	1	.034
Shrub heights	Treatment	41.77	1	.0001
	Year	12.76	1	.001

Linear‐mixed models with a Satterthwaite approximation for degrees of freedom were used with year as nested, random effect.

**Figure 4:**
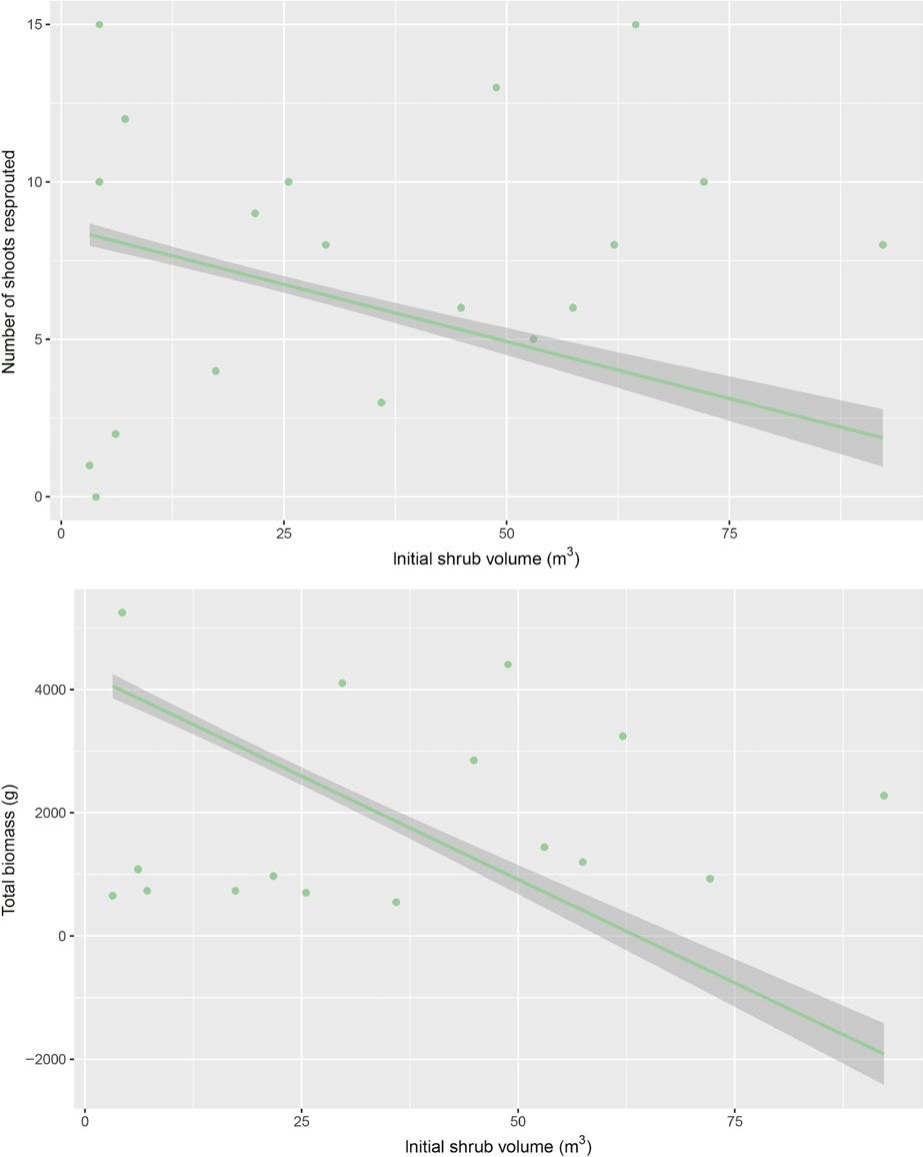
Regressions of the initial shrub size in 2013 prior to clipping, and the regrowth estimated by these shrubs at the end of the experimental period. Linear regressions were fit, and the estimates for number of shoots resprouted: *r* ^2 ^= .19, *p* = .0001 and for the total biomass: *r* ^2 ^= .4, *p* = .0001. The number of woody shoots that resprouted was counted in the field, shrubs were then reclipped to ground, and all aboveground biomass weighed to the nearest gram

Clipped, larger shrubs resprouted less and produced significantly less biomass after a two‐year regrowth period relative to smaller shrubs (Figure 4, linear regressions using initial shrub volume as predictor, number of shoots resprouted: *r* ^2 ^= .19, *p* = .0001 and total biomass: *r* ^2 ^= .4, *p* = .0001). Quantile regression analyses in both instances similarly modeled negative shrub recoveries with increasing size (*t* = −10.2, *p* = .0001 for shoots resprouted, and *t* = −11.9, *p* = .0001 for biomass).

## 4 DISCUSSION

Facilitation by shrubs in deserts is an increasingly established mechanism to restore and maintain deserts (Badano et al., 2016; Zhao et al., 2007) and semiarid ecosystems (Gómez‐Aparicio, 2009; Gomez‐Aparicio et al., 2004; Jankju, 2013; Noumi, Chaieb, Michalet, & Touzard, 2015). A recovery hypothesis for potential foundation plant species is thus a critical precursor to understanding the role that positive interactions play in human‐disturbed systems and needs to be tested in tandem with interaction studies. We colloquially term this hypothesis a Groot effect from the movie Guardians of the Galaxy 2 because a sentient woody tree, albeit small, resprouted following significant damage and functioned as a superhero. This shrub recovery hypothesis or benefactor/hero effect was supported for the shrub species *E. californica* in a highly fragmented desert ecosystem with some important caveats. This shrub clearly and consistently facilitates the abundance of other plant species but not necessarily richness. Mechanical clipping and removal effectively removes the canopy of a dominant desert shrub for a number of years. Recovery is viable for this species, but larger individual shrubs were not necessarily more resilient to damage. Importantly, this study also suggests a novel trait to consider in plant facilitation studies—the capacity for the foundation species to recover from significant physical damage.

The demonstration that a shrub species functions as a foundation species is not necessarily novel. Nonetheless, it is important to continue to advance the importance of positive interactions within community ecology more broadly (He, Bertness, & Altieri, 2013; Soliveres, Smit, & Maestre, 2014) by exploring whether new potential benefactor species have comparable effects or whether positive interactions are species specific (Callaway, 1998). *E. californica* is unique to the facilitation literature because it is a gnetophyte. Gnetophyta is a plant division with only three genera (*Gnetum, Welwitschia, and Ephedra*) and has been proposed to be the missing link between angiosperms and their evolutionary precursors (Bowe, Coat, & dePamphilis, 2000). In the most extensive syntheses on the nurse‐plant syndrome in stressful environments to date (Flores & Jurado, 2003; He et al., 2013; Maestre, Valladares, & Reynolds, 2005), only the genus *Ephedra* has been captured in three instances (and for two other species of *Ephedra* not the species herein) from a total of 642 reported benefactor species in deserts (Flores & Jurado, 2003). This is important because the trait set is unique with needle‐like leaves, slender woody stems, an extensive canopy, and no true flowers and fruits (Alfieri & Mottola, 1983). This morphology, lack of direct animal resource attractants, and its niche for arid environments are an opportunity to extend the scope of potential species relevant to conservation and restoration in deserts and for expansions to coevolutionary theory and phylogenetics (Valiente‐Banuet & Verdu, 2007). Another important general outcome of this study is that abundance but not necessarily richness of other plant species is enhanced within the plant community. Frequently, facilitation between plants is assumed to be a panacea for desirable restoration outcomes within stressful or disturbed environments (Lortie, 2017). Understanding the species specificity of foundation plant species is thus a relevant avenue of ecological research in deserts because the effects of this relatively unique shrub species aligned well with similar research suggesting that there is the capacity for functional equivalency of shrubs as benefactors.

A recovery hypothesis and test for a Groot Effect are important to basic and applied and plant community ecology for several reasons. Ecologically, addition or removal of a potential driver is a primary means to advance theory and do experiments. This is relatively uncommon within the sphere of positive interaction studies for the obvious ethical reason of introducing an extreme and likely fatal treatment to a predicted foundation species. In two experiments that used aboveground removal as a mechanism to explore the relative importance of a canopy, both instances clearly demonstrated that there is an interplay between direct and indirect effects of the canopy on other plant species (Holzapfel & Mahall, 1999; Michalet, Brooker, Lortie, Maalouf, & Pugnaire, 2015) but failed to test for a recovery effect. We propose that canopy removals, applied judiciously, can continue to be an effective tool to contrast the different mechanistic pathways associated with the facilitation of other plants such as shelter versus resources versus soil moisture or seed trapping (Filazzola & Lortie, 2014) and also functionally for beneficiary animals (Lortie, Filazzola, & Sotomayor, 2016) provided the recovery of the foundation species is also documented. This study supports two previous studies that directly and similarly examined desert shrub recovery (Martinez Carretero & Dalmasso, 2002; Vasek, Johnson, & Eslinger, 1975). In the first study, the response of two species of *Larrea* to clipping was examined in Argentina (Martinez Carretero & Dalmasso, 2002). *E. californica* recovery aligned well with this other study because predicted full recovery to clipping was possible for both shrub species, would take a number of years, and because shrub size, similarly estimated including canopy volumes, effectively predicted biomass. This is positive and compelling and suggests that desert shrubs can be resilient but that detailed research on type and extent of damage is needed. The second study examined foundation plant species following significant human disturbance associated with pipeline construction in a California desert (Vasek et al., 1975). *Larrea* was examined in detail, but *E. californica* was also reported as a species that natural revegetated the sites 12 years after a significant disturbance. These findings are nontrivial. Ecosystem function in high‐stress ecosystems can be anchored to foundation species, and estimates of extent of recovery of multiple basal species (such as many shrub species within a region) are needed (Angelini et al., 2011). Support for the recovery hypothesis in this study indicates that this is a viable and biologically meaningful measure to include in studies of positive interactions within high‐stress systems and that other representative basal plant species impacted by humans need to be tested.

Tests for size dependency are needed to inform theory and experimental designs that examine positive interactions. The positive ecological effects of *Ephedra* were not related to canopy sizes as tested in this single study suggesting that relatively smaller canopies and potentially younger individuals of this foundation species can nonetheless benefit the region. This is compelling evidence for a Groot Effect because it was a multiyear study and encompassed a period of extended regional drought. Similar desert research reported that increased shrub size was a negative driver on the abundance of associated forbs (Michalet et al., 2015), and a relevant meta‐analysis reported that “small shrubs” have significant and consistent positive effects on other plants including woody species (Gomez‐Aparicio et al., 2004). Nonetheless, shrub size can also have divergent effects with larger individuals more positively impacting community assembly, but this was dependent on the relativity aridity tested (Maestre & Cortina, 2005; Tewksbury & Lloyd, 2001). Hence, the mechanistic pathways associated with *Ephedra* facilitation were not size‐dependent, but other foundation species and the relative importance of seasonality can mediate these outcomes (and there was also significant variation in the effect of season on richness in this study further supporting this conclusion). Herein, recovery was not positively related to initial shrub sizes. This supports previous research that shows larger plants produce more dry matter following clipping but not disproportionately (Carretero & Dalmasso, 2002) and that smaller plants do not adversely respond to drought in deserts (Casper, Forseth, Kempenich, Seltzer, & Xavier, 2001). Research on resprouting after fire damage in arid systems is also not necessarily size‐dependent (Meyer, Ward, Moustakas, & Wiegand, 2005). Shrubs are thus a tractable experimental opportunity to examine fundamental theory such as facilitation and community assembly processes in high‐stress ecosystems. The capacity for large and small individuals of a foundation species to recover suggests that rehabilitation of desert remnants, within the San Joaquin Desert region in California for instance, is more likely to succeed and proceed more rapidly with an intact population of a shrub species present.

## CONFLICT OF INTEREST

None declared.

## AUTHOR CONTRIBUTION

CJL designed the experiment, did the statistics and visualizations, and wrote the manuscript. All co‐authors did the fieldwork and provided edits to the writing.
