# Supplementary material for: The Groot Effect: Plant facilitation and desert shrub regrowth following extensive damage
Source: Ecol Evol. 2017 Dec 5;8(1):706–15. doi: 10.1002/ece3.3671 (PMC5756850; doi:10.1002/ece3.3671)

**Appendix S1.** The regional climate data for Panoche Hills Ecological Reserve.


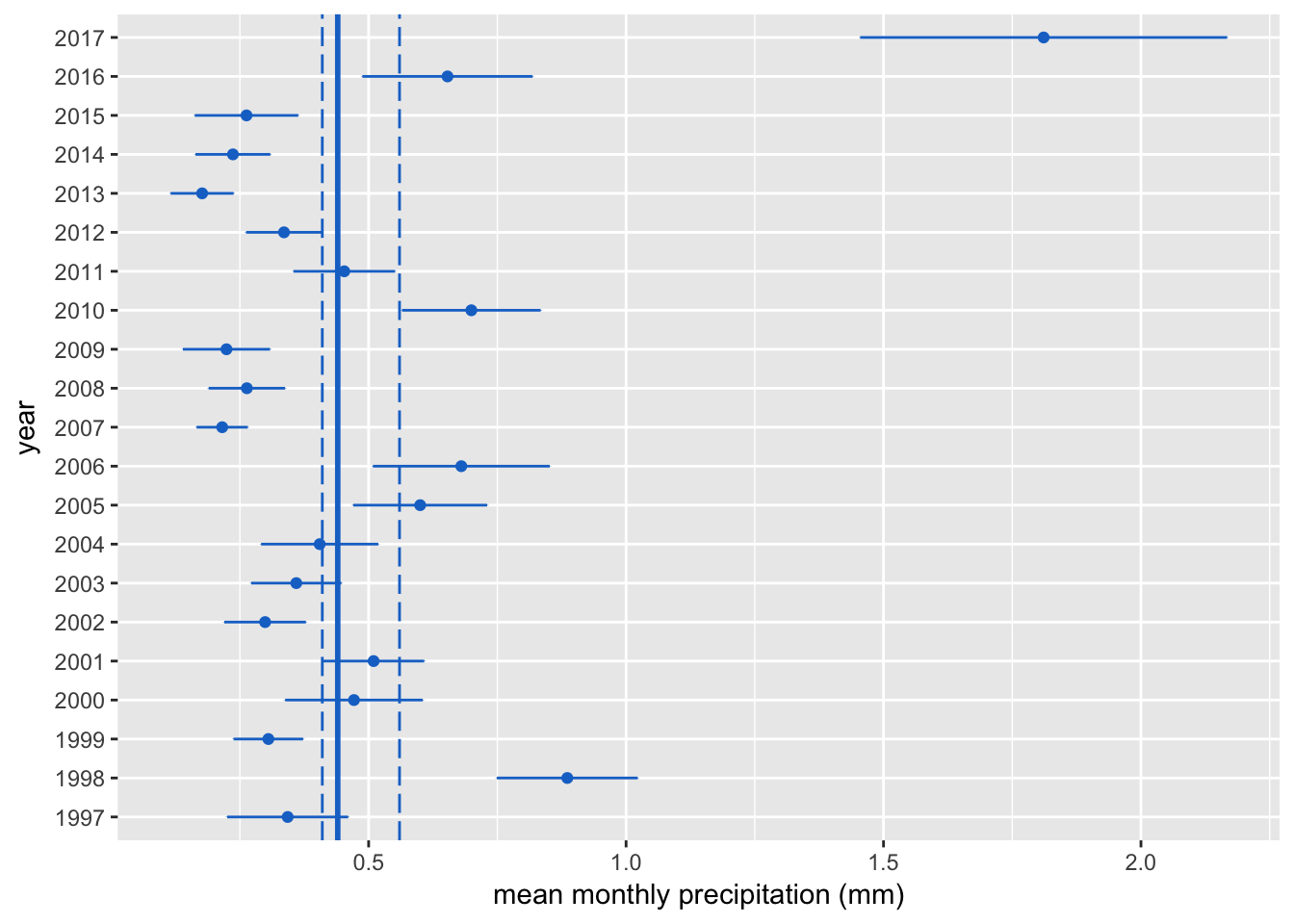


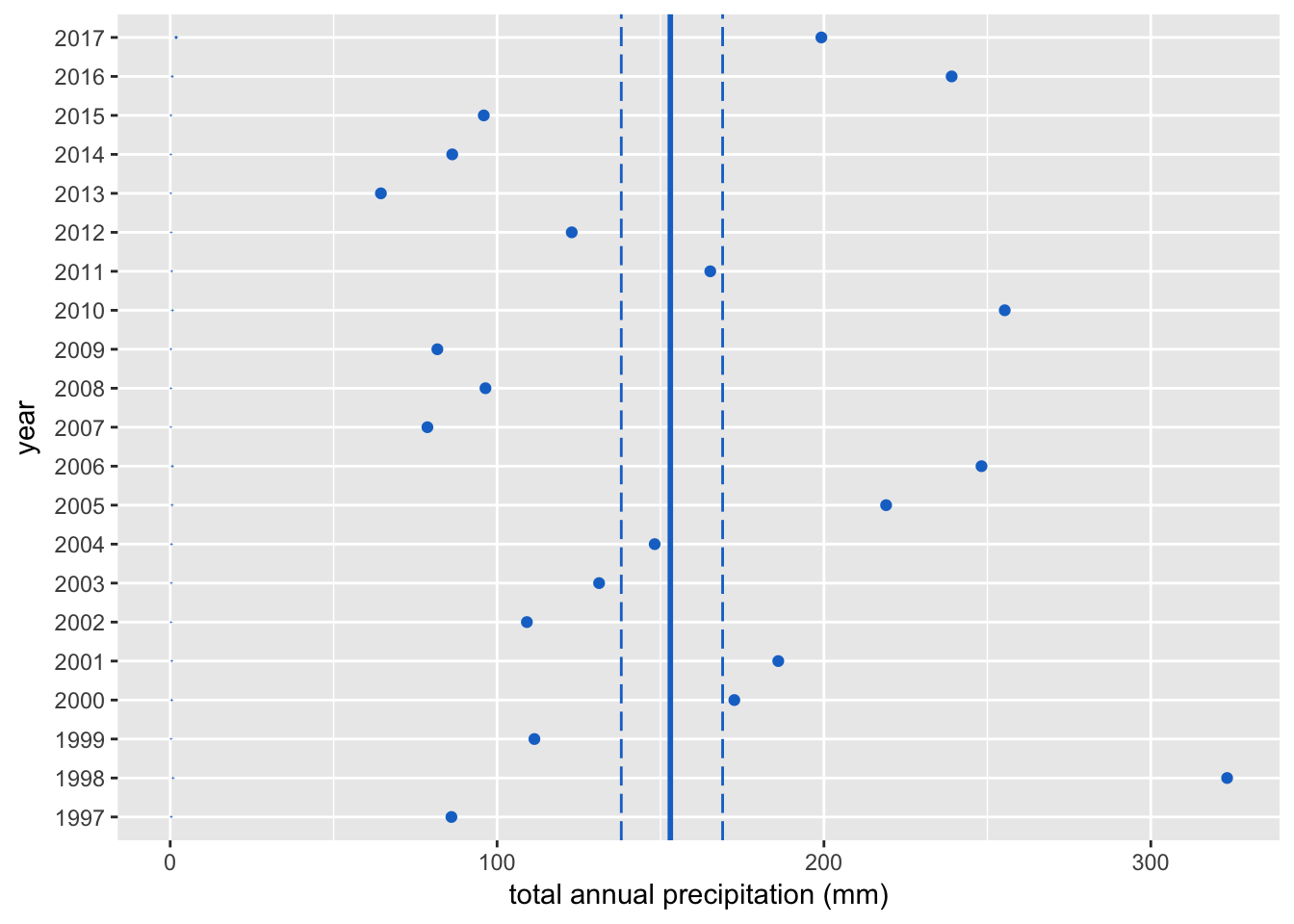


Climate data sourced from nearest weather station to Panoche Hills Ecological Reserve (http://ipm.ucanr.edu/calludt.cgi/WXSTATIONDATA?STN=PANOCHE.A). Full analyses and data wrangling provided here: <https://cjlortie.github.io/desert.climate.contrasts/>. Mean monthly precipitation points are shown with +/- 1 standard error estimated using the monthly values in a given year. The horizontal line in both plots is the 20-year mean (monthly and annual) precipitation for this site. The dotted lines are +/- 1 standard error around these long-term mean estimates.

The relative proportional differences between each mean monthly and total annual precipitation values were significantly different from the long-term estimates (One-sample t-tests, Mean monthly differences: t = 3.6115, df = 20, p-value = 0.001741, and Total annual precipitation differences: t = 7.3155, df = 20, p-value = 4.511e-07).

**Appendix S2.** The growth form of *Ephedra californica* is extremely ‘shrubby’ with extensive axillary branching and a very well developed canopy structure.

**
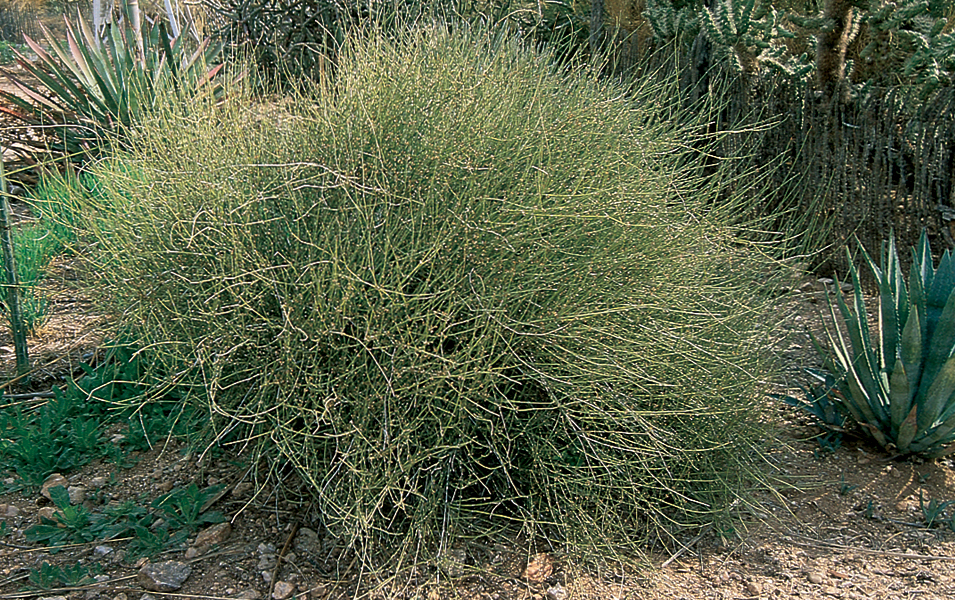
**

**Appendix S3.** The effect of *Ephedra californica* shrub volume estimates on the relative measures of the annual plant community (see Methods for full details).


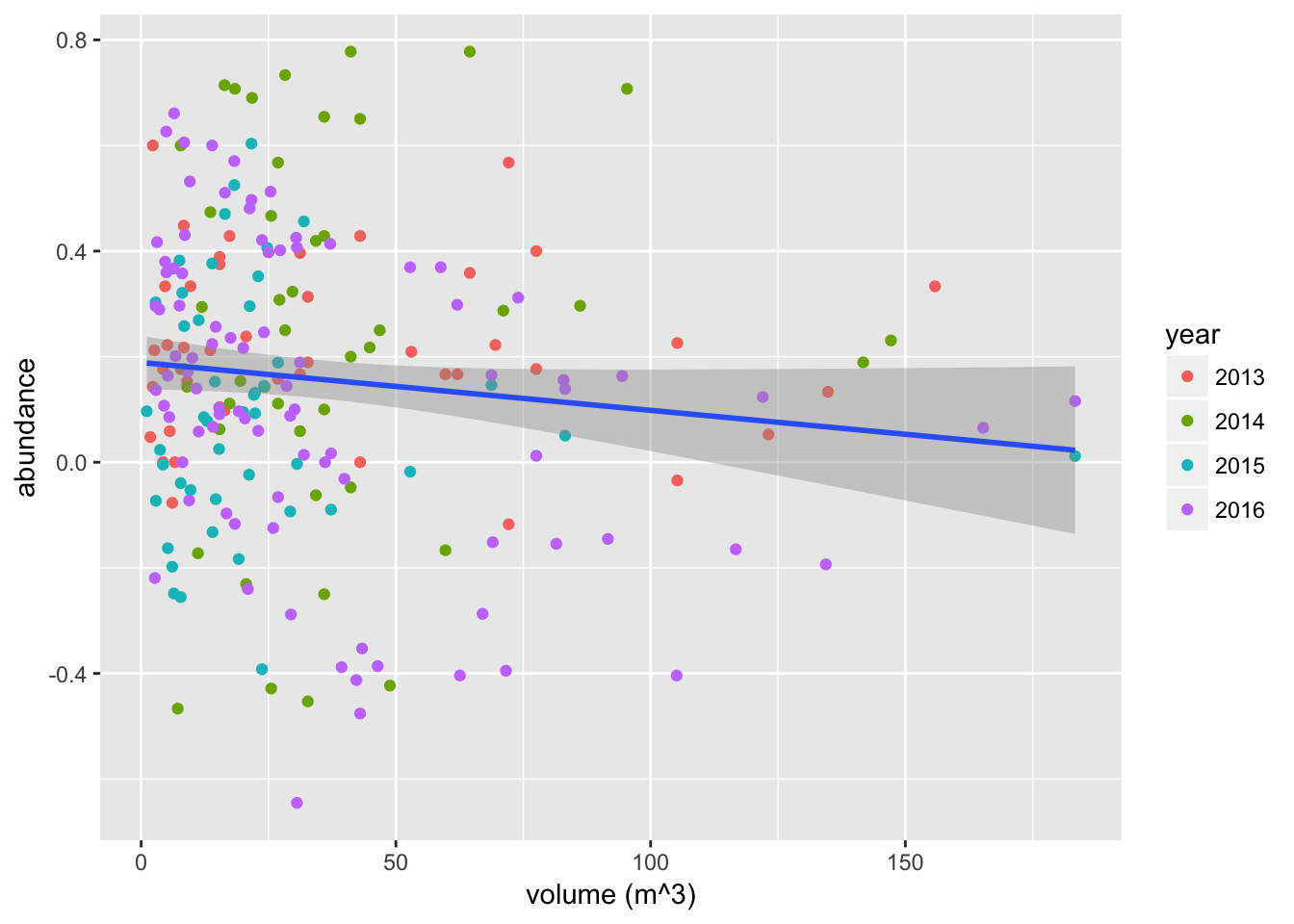


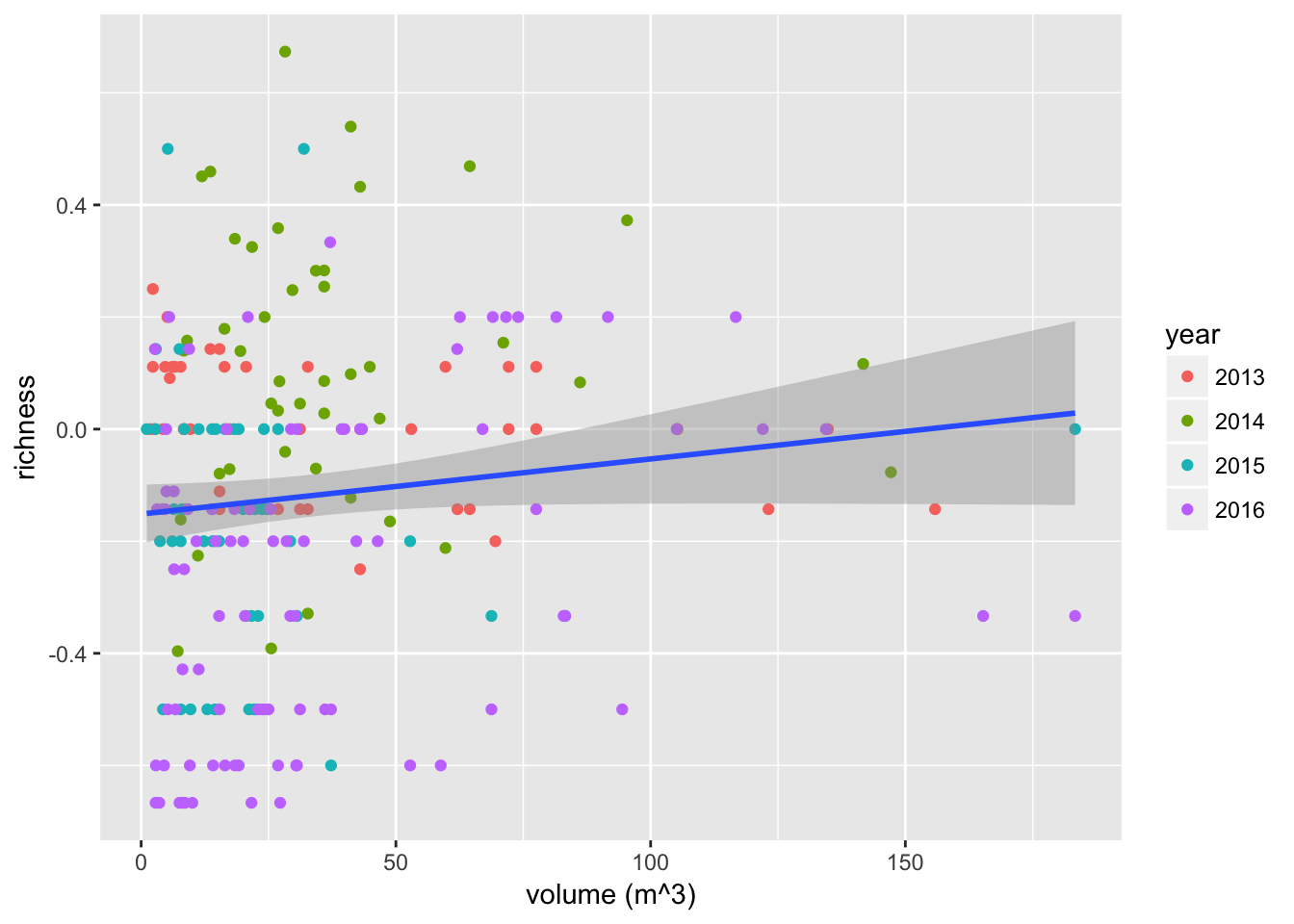


Linear regression analyses of shrub volumes on the annual plant community suggest that larger shrubs do not have significantly greater influence on the annual plant community measures tested in this study (regression analyses, abundance: r^2^ = 0.009, p = 0.08, df = 221; richness: r^2^ = 0.01, p = 0.07, df = 221). Quantile regression analyses for abundance similarly estimated a net negative slope not significantly different from 0 (t = -0.0003, p = 0.44,) whilst richness was estimated as positive using this method (t = 0.001, p = 0.013). In the latter instance, this does suggest that net volume predicts richness associated with shrubs statistically, but given the relatively small value of the coefficient for the slope and outcome of linear regression tests, we do not interpret this difference as ecologically meaningful. Each respective year sampled was also tested independently and similarly supports weak or inconsistent effects of shrub volumes on the net outcome of interactions with the annual plant community by year.


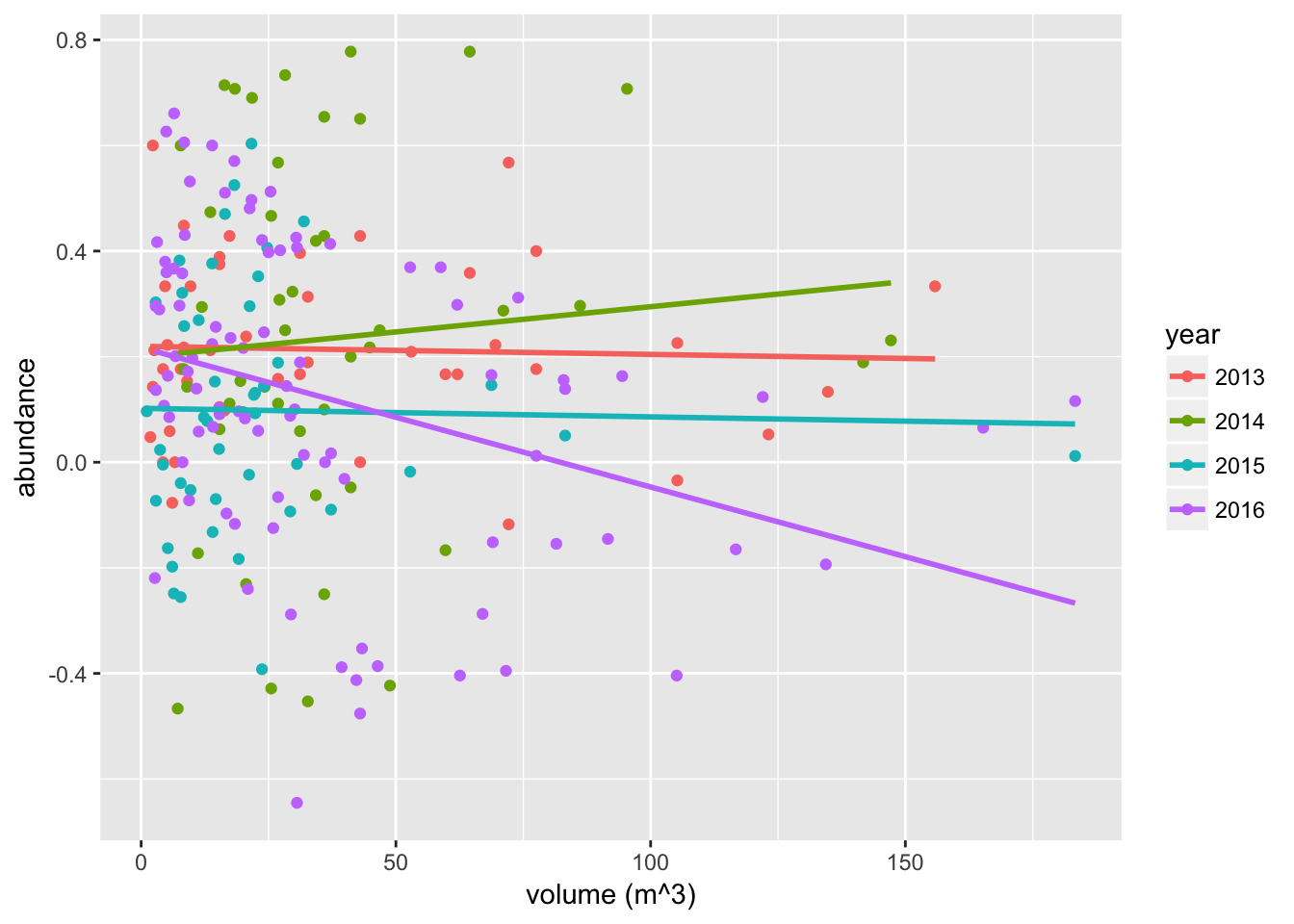


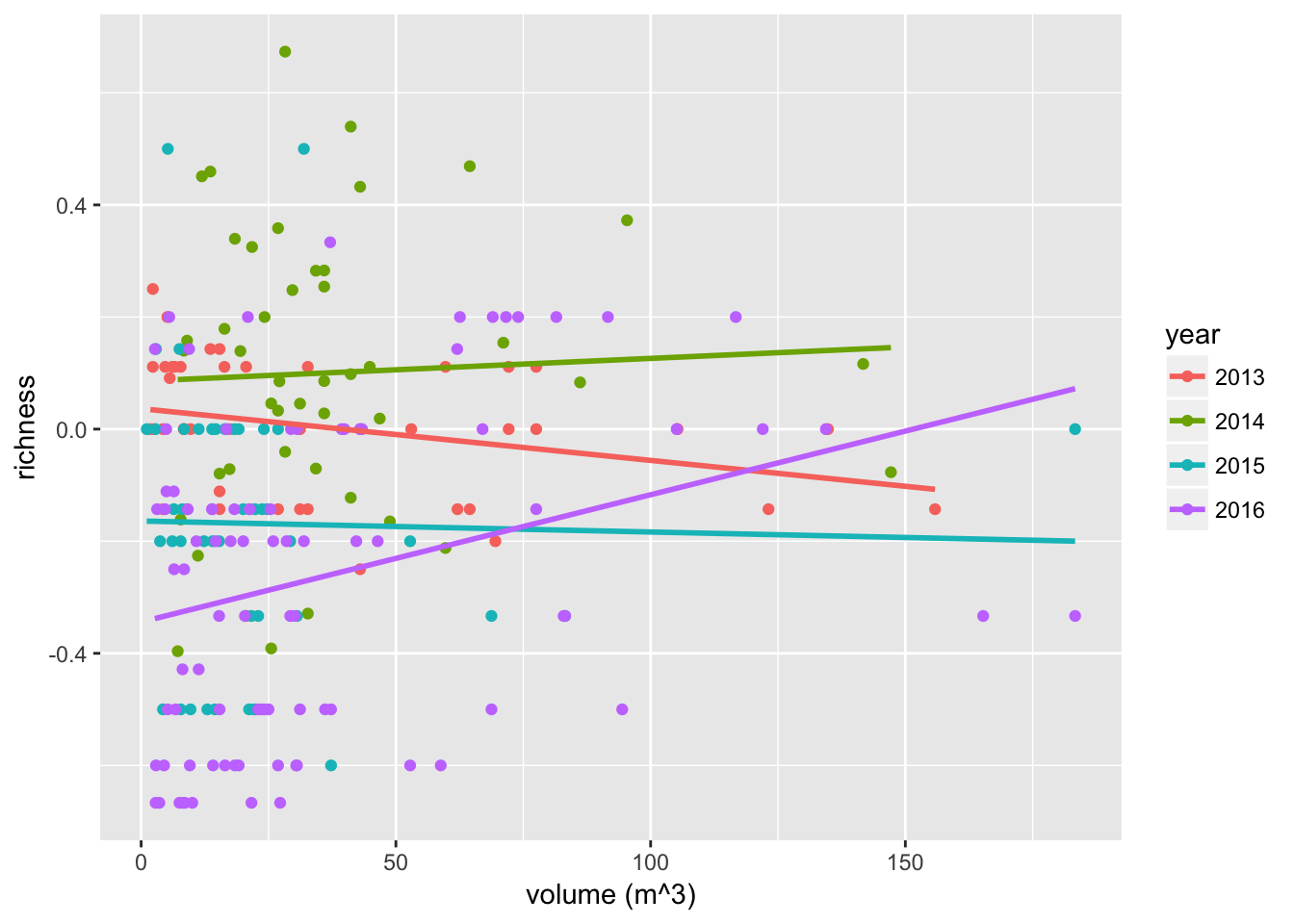

Supplement: Supplementary file 1 [file ECE3-8-706-s001.docx]
